# Oxidant/antioxidant imbalance is an inherent feature of depression

**DOI:** 10.1186/s12888-015-0454-5

**Published:** 2015-04-08

**Authors:** Monika Talarowska, Janusz Szemraj, Michael Berk, Michael Maes, Piotr Gałecki

**Affiliations:** 1 Department of Adult Psychiatry, Medical University of Lodz, Lodz, Poland; 2 Department of Medical Biochemistry, Medical University of Lodz, Lodz, Poland; 3 Orygen Research Centre, Parkville, Australia; 4 Department of Psychiatry, The University of Melbourne, Parkville, Australia; 5 Florey Institute for Neuroscience and Mental Health, Parkville, Australia; 6 IMPACT Strategic Research Centre, Deakin University, Geelong, Australia; 7 Psychiatry Department, Faculty of Medicine, Chulalongkorn University, Bangkok, Thailand; 8 Health Sciences Graduate Program, Health Sciences Center, State University of Londrina, Londrina, Brazil

**Keywords:** Depression episode, rDD, MnSOD, MPO, COX-2, i-NOS

## Abstract

**Background:**

50% to 60% of the people who have recovered from the first episode of depression experience a relapse. The immune system of the people suffering from depression is in a permanent state of pathological pro-inflammatory readiness. There are some reports that depressive episodes cause sensitization of immune-inflammatory pathways and that staing of depression (e.g. number of depressive episodes) is correlated with immune-inflammatory markers.

The main objective of the study was to delineate whether recurrent major depression (rDD) is characterized by alterations in selected immune-inflammatory biomarkers as compared with first episode of depression (ED-I), i.e. expression of mRNA and enzymatic activity of manganese superoxide dismutase (MnSOD, SOD-2), myeloperoxidase (MPO), inducible nitric oxide synthase (iNOS, NOS-2), and cyclooxygenase-2 (COX-2).

**Methods:**

The study was carried out in a group of 131 patients: ED-I group – 42 patients, rDD group – 89 patients. Depression severity was assessed with the 17-item Hamilton Depression Rating Scale (HDRS). The number of depression episodes and the disease duration periods were recorded in each patient. For the patients, HDRS was administered at admission during the symptomatic phase, which would generally be either before or shortly after modification of the previous antidepressant drug regimen. Reassessment of the mental condition was conducted after 8 weeks of the pharmacological treatment also with the use of the HDRS scale.

**Results:**

No significant statistical differences were found between the analysed groups as regards the intensity of depressive disorders. No differences in the expression of MnSOD, MPO, COX-2 and i-NOS genes on the level of both mRNA and protein were observed between both groups. No significant interrelation was noticed between the number of depression episodes experienced and the expression of selected genes on the mRNA level and protein level.

**Conclusions:**

There is no significant difference in MnSOD, MPO, COX-2 and i-NOS between patients with recurrent depressive disorders and those in a first episode of depression. These findings suggest that these enzymes are trait markers of depression and are not related to staging of depression.

## Background

Annual prevalence of depression in the adult population oscillates between 6% and 12%, and – according to different sources – varies from 5% to even 30% among people over the age of 65 [1]. Depression often accompanies other diseases, which means its symptoms are observed in approximately 10% of all adults within one year (this corresponds to 100 million cases).

Episodes of lower mood last relatively long – from 6 to 9 months. The disease may also take a chronic form, resistant to treatment. Age, at which the first episode takes place, as well as duration, frequency and intensity of episodes are characterised by individual variability [2].

50% to 60% of the people who have recovered from the first episode of depression experience a relapse. In the majority of hospitalised patients another depressive episode appears within the next two years of discharging from the hospital. It is estimated that some 20% of the affected with diagnosed recurrent depressive disorders experience two depressive stages during their life, and 60% – three or more such stages (3–4 on average) [3]. Every successive episode is associated with a less positive prognosis and poorer response to pharmacological treatment [4].

The first hypothesis that macrophages may play a role in depression was published in 1991 by Ronald Smith [5], whereas the first original reports on activation of immune-inflammatory pathways in depression were reported as of 1990 by Maes et al. Today, it is agreed that the immune system of the people suffering from depression is in a permanent state of pathological pro-inflammatory readiness [6]. The symptoms of an ongoing inflammatory process, like fatigue, sleep disorders, anxiety, low mood, loss of appetite or anhedonia, correspond to the symptoms of depressive disorders [7,8]. On the other hand, not only proinflammatory cytokines, e.g. tumor necrosis factor-alpha – (TNF-α), the interleukins (IL) and interferon-gamma (IFN-gamma), but also anti-inflammatory cytokines are released by CNS and peripheral-derived immune cells and play a powerful role in depression [9]. There are data that depressive episodes are accompanied by an increased sensitization of immune-inflammatory pathways and that the number of depressive episodes is correlated with immune-inflammatory markers such as TNF-α and neopterin [10].

The main objective of the study was to examine whether recurrent major depression (rDD) is accompanied by more profound inflammatory disturbances than the first episode of depression (ED-I), i.e. expression of mRNA and protein levels of four inflammatory enzymes, i.e. manganese superoxide dismutase (MnSOD, SOD-2), myeloperoxidase (MPO), inducible nitric oxide synthase (iNOS, NOS-2), and cyclooxygenase-2 (COX-2). Not only do the aforementioned compounds take part in an inflammatory reaction, but they are also active in the production of free radicals, and damage proteins, fatty acids and cellular DNA [11,12]. In our previous studies, we demonstrated that the selected variables (COX-2, iNOS, MnSOD, MPO) can have a significant impact on cognitive functioning in patients with rDD. Therefore, we have made an attempt to assess the level of selected indicators at different stages of the disease (rDD).

### Subjects

The study was carried out in a group of 131 patients: ED-I group – 42 patients, rDD group – 89 patients. All patients were hospitalised at Department of Adult Psychiatry Medical University of Lodz (Poland). The selection of individuals for the study group was performed randomly without replacement sampling.

The patients were selected for the study based on the inclusion criteria for ED and rDD outlined in ICD-10 (F32.0-7.32.2, F33.0-F33.8) [13]. The presence of axis I and II disorders, other than depressive episode, and the diagnosis of somatic diseases and injuries of the central nervous system (CNS), were regarded as exclusion criteria. Other exclusion criteria were: inflammatory or autoimmune disorders and unwilling to give informed consent. For all the subjects, a case history was obtained prior to participation using the standardised Composite International Diagnostic Interview (CIDI) [14].

All the subjects were free from medical illnesses, including infections and inflammatory or allergic reactions. None of the control subjects or depressed patients was treated with drugs known to influence lipid metabolism, immune response or endocrine function. None of the participants were drinkers or heavy smokers, and none had ever taken psychotropic drugs.

## Methods

### Severity of depression

Depression severity was assessed with the 17-item Hamilton Depression Rating Scale (HDRS). A description of HDRS has been presented elsewhere [15,16].

All the subjects were examined during the course of their hospitalisation. The study group included subjects, hospitalised for the first time for depressive episode and depression treatment-naïve, as well as those, treated for many years before and with multiple hospitalisation episodes in history, the latter admitted for various degrees of health deterioration. The number of depression episodes and the disease duration periods were recorded in each patient.

For the patients, HDRS was administered at admission during the symptomatic phase, which would generally be either before or shortly after modification of the previous antidepressant drug regimen. Reassessment of the mental condition was conducted after 8 weeks of the pharmacological treatment also with the use of the HDRS scale. Examination of patients was conducted by the same person in each case.

Whole blood samples from the patients were collected in 5 ml EDTA-containing tubes than centrifuge at 1000xg for 10 min at 4 degrees C and used for isolation peripherial blood lymphocytes. Lymphocytes and serum stored at – 70°C until analysed

### mRNA and serum protein expression

#### COX-2 mRNA and serum protein expression

Analyses were performed according to methods previously described: mRNA expression in the peripheral blood lymphocytes was quantified by real-time PCR using ABI Prism 7000 Sequence Detection System (Applied Biosystems, Foster City, Calif., USA) according to the manufacturer’s protocol [17], serum protein level by ELISA method (COX-2 ELISA Kit, Calbiochem, Merck KGaA, Darmstadt, Germany).

### iNOS mRNA and serum protein expression [18]

Nitric oxide was estimated in plasma with the use of Nitric Oxide Non-Enzymatic Assay (cat. No. 12111) Oxis International, Inc. Bioxytech.

Concentration of NO was measured in terms of its products nitrite (NO2-) and nitrate (NO3-). The test is based on the reduction of nitrate to nitrite using the granulated cadmium and conversion, using Greiss reagent, of nitrite into a purple compound – chromophore, which is measured colometrically at 540 nm. NO products are expressed as μM/L of plasma. The human iNOS and GADPH expression the peripheral blood lymphocytes was quantified by real-time PCR using ABI Prism 7000 Sequence Detection System (Applied Biosystems, Foster City, Calif., USA) according to the manufacturer’s protocol [18].

### MnSOD mRNA and serum protein expression

The test protein levels were measured in blood serum levels for each patient. These tests were preceded by a determination of serum total protein. Two measurement techniques were employed [19]. For the quantitative detection of serum SOD2 protein levels, a commercial kit, NWLSSTM MnSOD ELISA, was used (Northwest Life Science Specialties LLC, Vancouver, Wash., USA). The human MnSOD and GADPH expression the peripheral blood lymphocytes was quantified by real-time PCR using ABI Prism 7000 Sequence Detection System (Applied Biosystems, Foster City, Calif., USA) according to the manufacturer’s protocol [19].

### MPO mRNA and serum protein expression

For the quantitative detection of circulating serum MPO protein level, the commercial Human MPO Immunoassay from R&D Systems, Inc. (Minneapolis, MN, USA), was used. Total RNA (1 μg) was extracted from the the peripheral blood lymphocytes using Trizol reagent (Life Technologies Inc.), and was processed directly to cDNA synthesis using the TaqMan Reverse Transcription Reagents kit (Applied Biosystem) according to the manufacturer’s protocol [20].

### Statistical analysis

A statistical analysis of the collected material included calculation of both descriptive and inferential statistics. A two-tailed critical region was employed in the statistical hypothesis testing.

Qualitative characteristics of the experimental and control groups were expressed as frequencies shown as percentages. To characterise the average values for quantitative features, the arithmetical mean (M) and median (Me) were calculated. The measures of statistical dispersion included the range of values between the minimum and the maximum, and the standard deviation (SD).

Distributions were analysed using the Shapiro-Wilk test. To compare nonparametric variables in the test groups, the following tests were used: the Pearson *χ*2 for qualitative variables, the Wilcoxon signed-rank test for two related groups for quantitative variables, and the Mann–Whitney *U* test for two independent groups to determine the coincidence of distributions. To evaluate the relations between the analysed variables, Spearman's R rank order correlation coefficients were estimated. For all analyses, statistical significance was defined as p <0.05 [21]. All data analyses were performed using STATISTICA PL, version 10.

### Ethics

Before deciding to participate in the study, the subjects were informed of the purpose of the study, assured for voluntary participation, and guaranteed with personal data confidentiality. Written informed consent was obtained according to the study protocol that was approved by the Bioethical Committee of the Medical University of Lodz (No. RNN/728/12/KB).

## Results

Average age of all the examined patients (*N* = 131) was: *M* = 48.53 years, *SD* = 11.05; minimum age – 20 years, maximum – 67 years. In the ED-I group, average age was: *M* = 44.72, *SD* = 13.03, and in the rDD group: *M* = 49.89, *SD* = 9.68. The characteristics of the examined group in Table 1.

**Table 1:** **The comparison of the study groups in terms of sex and education**

Sex	ED-I ( *N* = 42)	rDD ( *N* = 89)	Total
	N (%)	N (%)	N (%)
Female	19 (45.24)	57 (64.04)	76 (58.01)
Male	23 (54.76)	32 (35.96)	55 (41.98)
**Education**	**ED-I** (*N* = 42)	**rDD** (*N* = 89)	**Total**
	N (%)	N (%)	N (%)
Primary	1 (2.38)	9 (10.11)	10 (7.63)
Vocational	11 (26.19)	18 (20.22)	29 (22.14)
Secondary	20 (47.62)	46 (51.69)	66 (50.38)
High	10 (23.81)	16 (17.98)	26 (19.85)

*ED-I – first episode of depression, rDD – recurrent depressive disorders, n – number of samples, % – percentage.*

No significant statistical differences were found between the examined groups in terms of sex (*χ2* = 4.14, *p* = 0.41) and education (*χ2* = 3.27, *p* = 0.35), only in terms of age (*Z* = 2.21, *p* = 0.03).

Significant statistical differences were observed between severity of depression as measured with the HDRS scale in the ED-I and rDD groups on the day of admission to the experiment and after obtaining a response to the applied pharmacological treatment (respectively *Z* = 5.64, *p* < 0.001 and *Z* = 8.18, *p* < 0.001). This result indicates an improvement in the effect of the incorporated treatment in both the examined groups.

No significant statistical differences were found between the analysed groups as regards the severity of depressive disorders (Table 2). Neither were such differences observed on the day of admission of the patients to the experiment as well as after receiving a response to the implemented pharmacological treatment. In both groups, average level of the symptoms of depressive disorders on the first day of the experiment corresponded to severe level of depressive disorders acc. to the HDRS scale and remission of depressive disorders according the HDRS scale after 8 weeks of the pharmacological therapy.

**Table 2:** **The severity of depressive disorders among ED-I group and rDD group**

Variable	ED-I ( *N* = 42)	rDD ( *N* = 89)	Mann–Whitney *U* test	
	M (SD)	M (SD)	Z	p
HDRS-I	22.73 (6.51)	24.06 (6.59)	−0.622	0.534
HDRS-II	5.62 (3.76)	6.86 (4.01)	−1.53	0.126
Number of depression episodes	-	6.48 (5.81)	-	-

*ED-I – first episode of depression, rDD-recurrent depressive disorders, HDRS-I – Hamilton Depression Rating Scale at the onset of therapy, HDRS-II – Hamilton Depression Rating Scale after pharmacological treatment, M – mean, SD – standard deviation.*

Table 3 presents average values, standard deviation, minimum and maximum values of expression on the mRNA level and the protein level for the analysed inflammation enzymes: MnSOD, MPO, COX-2 and iNOS in the examined group (*N* = 131).

**Table 3:** **Average standard deviation, minimum and maximum values of expression of selected genes on the mRNA level and protein level in the examined group (*N*** **= 131)**

Variable	rDD		
	M (SD)	Min.	Max.
MnSOD	0.54	0.21	0.83
mRNA (2^-ΔΔct^)	(0.17)		
MnSOD	175.37	67	294
protein (pg/ml)	(64.74)		
MPO	0.31	0.23	0.43
mRNA (2^-ΔΔct^)	(0.04)		
MPO	308.95	219	421
protein (ng/ml)	(39.94)		
COX-2	0.24	0.13	0.33
mRNA (2^-ΔΔct^)	(0.04)		
COX-2	114.66	47	212
protein (ng/ml)	(24.67)		
iNOS	0.11	0.05	0.18
mRNA (2^-ΔΔct^)	(0.02)		
iNOS	20.71	4.91	43.61
protein (U/ml)	(8.19)		

*MnSOD – manganese superoxide dismutase, MPO – myeloperoxidase, COX-2 – cyclooxygenase-2, iNOS – cytokine-inducible nitric oxide synthase, M – mean, SD – standard deviation.*

Table 4 presents a comparison of expression of the analysed genes on the mRNA level and protein level in the affected from the ED-I and rDD group.

**Table 4:** **Comparison of expression of the analysed genes on the mRNA level and protein level in the affected from the ED-I and rDD group**

Variable	ED-I	rDD	Mann–Whitney *U* test	
	M (SD)	M (SD)	Z	p
MnSOD	0.529	0.548	0.266	0.791
mRNA (2^-ΔΔct^)	(0.189)	(0.158)		
MnSOD	172.26	176.83	0.283	0.776
protein (pg/ml)	(72.78)	(60.97)		
MPO	0.319	0.314	−0.531	0.596
mRNA (2^-ΔΔct^)	(0.037)	(0.041)		
MPO	311.16	307.91	−0.271	0.786
protein (ng/ml)	(38.08)	(40.96)		
COX-2	0.242	0.235	−0.806	0.421
mRNA (2^-ΔΔct^)	(0.039)	(0.038)		
COX-2	116.47	113.79	−0.483	0.628
protein (ng/ml)	(24.84)	(24.71)		
iNOS	0.103	0.101	−0.086	0.931
mRNA (2^-ΔΔct^)	(0.023)	(0.022)		
iNOS	21.01	20.56	0.044	0.964
protein (U/ml)	(8.66)	(8.02)		

*ED-I – first episode of depression, rDD – recurrent depressive disorder, MnSOD – manganese superoxide dismutase, MPO – myeloperoxidase, COX-2 – cyclooxygenase-2, iNOS – cytokine-inducible nitric oxide synthase, M – mean, SD – standard deviation.*

In case of all the variables included in the analysis, no significant statistical differences were found between the ED-I and rDD group. No differences in the expression of MnSOD, MPO, COX-2 and i-NOS genes on the level of both mRNA and protein were observed between the patients with the first episode of depression and diagnosed with rDD.

An average number of depression episodes totalled 6.48 among the patients suffering from rDD (Table 2). No significant interrelation was noticed between the number of depression episodes experienced and the expression of selected genes on the mRNA level and protein level.

In the examined group, there was no significant relationship between the severity of depressive disorders measured before and after pharmacotherapy and the expression on the mRNA level and protein level for the analysed genes.

## Discussion

The working hypothesis presented in the introduction was not confirmed. The obtained results indicate no differences in the expression of inflammation enzymes (MnSOD, MPO, COX-2 and iNOS) between patients with the first episode of depression and rDD. However, in our previous experiments we showed that the expression of mRNA and enzyme activity of MnSOD was significantly lower in rDD patients than in controls [19], a phenomenon which could point toward an adaptive MnSOD response to inflammation and increased oxidative stress. Previously, we have shown that myeloperoxidase [20], cyclooxygenase-2 [17] and inducible nitric oxide synthase [18] mRNA expression and protein levels were significantly higher in patients with recurrent depressive disorders than in healthy controls. Therefore, the results may indicate actively progressing immune-inflammatory and oxidative stress processes in patients with depression, although the magnitude of biomarkers measured here does not depend of the phase of the disease Table 5.

**Table 5:** **The significance of inflammation enzymes in the aetiology of depression**

MnSOD	First line of defence against damage caused as a result of excessive production of the superoxide anion radical by the mitochondrion [9]. Experiments of recent years indicate that MnSOD protects cells of the CA1 region of the hippocampus against apoptosis [33]. Additionally, MnSOD also protects cells of the CA3 region of the hippocampus against generation of free radicals during excessive glutamatergic activity [34].
MPO	Protein of an immuno-inflammatory response and prooxidative enzyme, which may be a marker of inflammation as well as prooxidative processes in the patients with depression [35]. MPO is a heme enzyme whose expression is observed in both cells of the brain and immune cells, which indicates its crucial role in the regulation of inflammatory processes and the formation of oxidative stress [36]. MPO activation leads to the production of hypochlorous acid and other toxic oxidising agents [37]. According to Vaccarino et al. [23], a raised level of MPO may be considered a biomarker of inflammation in the course of rDD.
COX-2	COX-2 inhibition restricts the formation of reactive oxygen species [38]. COX-2 also affects an increase of activity in the hypothalamus – pituitary gland – adrenal glands axis, and an increase of the level of pro-inflammatory cytokines [39].
iNOS	Plays a decisive role not only in numerous biological processes, but also in the regulation of cognitive and emotional functions, which may be significant in the aetiology of anxiety and depressive disorders (most of all through participation in neuromodulation, neurotransmission and synaptic plasticity) [40,41]. Nitrous oxide – being one of free radicals – takes part in the regulation of oxidative stress [12]. NO plays a neuroprotective role in physiological conditions. Nevertheless, if produced in excess, or if cells are under the influence of oxidative stress, the action of nitrous oxide becomes harmful; then, it undergoes oxidation and reduction processes producing toxic compounds referred to as reactive nitrogen species (RNS), which cause cell damage. Both NO and RNS play a role in the pathogenesis and development of many neurodegenerative diseases [42].

*MnSOD – manganese superoxide dismutase, MPO – myeloperoxidase, COX-2 – cyclooxygenase-2, iNOS – cytokine-inducible nitric oxide synthase.*

There are no studies which have compared the biomarkers we have used between both depression groups and therefore we cannot compare our results with the reports of other researchers. However, referring to the work by Sarapas et al. [22] we shall use two expressions in this place – the so-called state effects and trait-like relationship. According to the first hypothesis, an increase of inflammation factors observed during an episode of depression is only a transient condition, which disappears in the period of disease remission. Based on the second hypothesis, increased pro-inflammatory and anti-inflammatory activation is a permanent feature accompanying patients who suffer from depression. The present results show that alterations in the enzymes measured here are trait markers, whereas previous research showed that increases in TNF-α and neopterin should be regarded as staging markers [10]. In any case, it seems justified to conclude that some activated immune-inflammatory and oxidative stress pathways may be considered as endophenotypic trait biomarkers of depression, whereas other assays are biomarkers of staging of depression.

In the examined group, there were no significant relationships between the severity of depressive disorders measured before and after pharmacological treatment and the expression of mRNA and protein levels of the enzymes. Moreover, the relationship between rDD level and MPO concentration in blood serum was not confirmed by Vaccarino et al. [23]. Meanwhile, in the experiments conducted by Sarandol et al. [24], a raised level of SOD-2 in the group of patients with the so-called major depressive disorder positively correlated with the intensity of symptoms of depressive disorders. Similar conclusions were drawn from the work by Su et al. [25], who evaluated the concentration of inflammatory markers such as IL-6 and C-reactive protein. Additionally, it was demonstrated that increased concentration of IL-2 correlated with the risk of an attempted suicide [26].

Antidepressant treatments may reduce oxidative stress [27-29]. However, Gałecki et al. [30] did not find a change in the concentration of antioxidative enzymes (catalase and zinc superoxide dismutase) after 3 months fluoxetine pharmacotherapy in patients with rDD (*n* = 50). The concentration of the said compounds – both before onset of the treatment and after observing a symptomatic improvement in the examined group of patients with rDD – was higher than in healthy individuals. In another paper [31], a desired outcome (concentration reduction of catalase and zinc superoxide dismutase in serum) was possible after combining fluoxetine-based pharmacotherapy with acetylsalicylic acid (non-steroid anti-inflammatory drug). Similar results were recorded by Whittle et al. [32] in an experiment based on an animal model.

In summary, it is possible to confirm the necessity for further research studies as regards the presented issues and mutual interrelations between inflammatory processes and emotional and cognitive signs of depression.

## Conclusions

There is no significant difference in MnSOD, MPO, COX-2 and i-NOS between patients with recurrent depressive disorders and those in a first episode of depression. These findings suggest that these enzymes are trait markers of depression and are not related to staging of depression.

## Abbreviations

CIDI: Composite international diagnostic interview
COX-2: Cyclooxygenase-2
ED-I: First episode of depression
HDRS: Hamilton depression rating scale
IFN-gamma: Interferon-gamma
IL: Interleukins
iNOS, NOS-2: Inducible nitric oxide synthase
MNSOD, SOD2: Manganese superoxide dismutase
MPO: Myeloperoxidase
rDD: Recurrent depressive disorder
TNF-α: Tumor necrosis factor-alpha
